# Next-Generation Influenza HA Immunogens and Adjuvants in Pursuit of a Broadly Protective Vaccine

**DOI:** 10.3390/v13040546

**Published:** 2021-03-24

**Authors:** Kaito A. Nagashima, Jarrod J. Mousa

**Affiliations:** 1 Center for Vaccines and Immunology, College of Veterinary Medicine, University of Georgia, Athens, GA 30602, USA; kaito.nagashima25@uga.edu; 2 Department of Infectious Diseases, College of Veterinary Medicine, University of Georgia, Athens, GA 30602, USA

**Keywords:** influenza, vaccine, universal influenza vaccine, adjuvant, antibodies, hemagglutinin, broadly neutralizing antibodies

## Abstract

Influenza virus, a highly mutable respiratory pathogen, causes significant disease nearly every year. Current vaccines are designed to protect against circulating influenza strains of a given season. However, mismatches between vaccine strains and circulating strains, as well as inferior vaccine effectiveness in immunodeficient populations, represent major obstacles. In an effort to expand the breadth of protection elicited by influenza vaccination, one of the major surface glycoproteins, hemagglutinin (HA), has been modified to develop immunogens that display conserved regions from multiple viruses or elicit a highly polyclonal antibody response to broaden protection. These approaches, which target either the head or the stalk domain of HA, or both domains, have shown promise in recent preclinical and clinical studies. Furthermore, the role of adjuvants in bolstering the robustness of the humoral response has been studied, and their effects on the vaccine-elicited antibody repertoire are currently being investigated. This review will discuss the progress made in the universal influenza vaccine field with respect to influenza A viruses from the perspectives of both antigen and adjuvant, with a focus on the elicitation of broadly neutralizing antibodies.

## 1. Introduction

Influenza virus is a major cause of respiratory disease, causing significant morbidity and mortality in the United States and across the globe. Influenza epidemics typically occur annually, with estimates attributing between 9.3 and 38 million illnesses, and between 140,000 and 810,000 hospitalizations in the United States, to the disease each year [1]. Importantly, the detection incidence of influenza virus has diminished since the implementation of community mitigation strategies to prevent the spread of SARS-CoV-2, although external factors including viral interference may play a role [2]. Influenza virus is a negative-sense, segmented, single-stranded RNA virus of the family *Orthomyxoviridae*, and is categorized into four genera: A, B, C, and D [3]. The predominant influenza genera, A and B, are further stratified into phylogenetic groups and lineages. Influenza A viruses (IAVs) are categorized into two broad groups based on the relative differences of the hemagglutinin (HA) protein: group 1 and group 2; group 1 consists of subtypes H1, H2, H5, H6, H8, H9, H11, H12, H13, H16, H17, and H18, and group 2 of subtypes H3, H4, H7, H10, H14, and H15 [4]. Influenza B viruses consist of virus strains of the Victoria and Yamagata lineages, which co-circulate and demonstrate plasticity in the HA and neuraminidase (NA) proteins due to immune pressure [5]. Vaccination is effective at reducing the incidence of influenza virus, but annual vaccination is required for inducing protection due to constant antigenic drift. In efforts to obviate the necessity for yearly vaccines, and to increase their effectiveness against circulating strains, several approaches towards ‘universal’ influenza vaccines, those that elicit an immune response against the majority of encountered influenza viruses, have been pursued. Furthermore, the mechanisms of adjuvants have garnered attention in an effort to improve the immune response induced by such influenza immunogens. In this review, HA-based approaches to universal influenza vaccines will be discussed, with a focus on IAVs due to their higher diversity. Given that adjuvants have been recognized as a key component of influenza vaccines by conferring a robust immune response, a number of adjuvants under study will also be discussed.

## 2. Humoral Immune Responses to Influenza

The humoral immune response to influenza infection and vaccination comprises an essential part of host defense. Antibodies targeting the two predominant viral surface glycoproteins, HA and NA, neutralize influenza virus by inhibiting viral attachment/fusion and release, respectively. Moreover, hemagglutination/HA inhibiting (HAI) titers and NA inhibition (NAI) titers are important correlates of protection [6]. HA is responsible for viral attachment to sialylated host cell receptors, as well as entry through fusion with the endosomal membrane during the course of infection. The HA protein of human-tropic strains preferentially recognizes α(2,6)-linked sialic acid, while avian-tropic strains utilize α(2,3)-linked sialic acid [7]. Expression of α(2,3)-linked sialic acid differs across avian species [8]. Within a species, tissues within the respiratory and gastrointestinal tracts exhibit differential sialic acid expression that permit viral attachment [8]. Species with avian and human receptors, such as quail, turkey, and pigs, may permit zoonosis [8]. HA evolution mediates the adaptation of avian-origin strains to replicate in humans. The amino acid residues within the receptor-binding site (RBS) of HA favor either human or avian tropism. In H9N2 avian influenza virus isolates, a glutamine at position 226 within the RBS is known to favor human-type α(2,6)-linked sialic acid receptors [9]. Furthermore, a non-RBS residue at position 190, which affects viral binding affinity to murine and human lung-expressed sialic acid, is implicated in the initial stages of viral replication [9].

**Figure 1:**
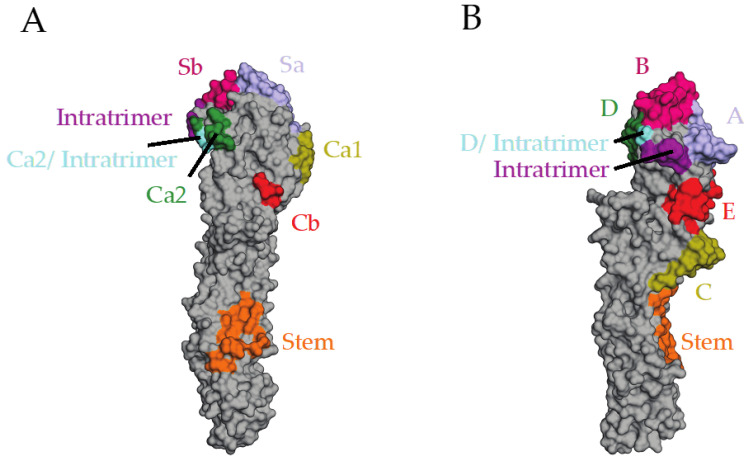
Antigenic sites and epitopes on H1 and H3 hemagglutinins (HAs). (**A**) H1 antigenic sites and antibody epitopes labeled on the A/California/04/2009 HA (gray). Antigenic sites Sa (light blue), Sb (magenta), Ca1 (dark yellow), Ca2 (green), and Cb (red) are located on the globular head domain. The intratrimeric epitope, represented by the FluA-20 antibody epitope (purple), is present on the interface between HA protomers in the trimer. Residues overlapping the intratrimeric epitope and the Ca2 antigenic site are in sky blue. Sa, Sb, and Ca2 comprise the periphery of the receptor-binding site (RBS). The epitope of a H1 stem-reactive antibody, CR6261, is shown in orange. (**B**) H3 antigenic sites and antibody epitopes on the A/Hong Kong/1/1968 HA (gray). Antigenic sites A (light blue), B (magenta), C (dark yellow), D (green), and E (red) are present on the head, as is the intratrimeric epitope (purple), shown as the epitope of FluA-20. Residues in both antigenic site D and the intratrimeric epitope are shown in sky blue. Antigenic sites A, B, and D comprise the RBS. The epitope of a stem antibody reactive to H3 viruses, CR9114, is labeled in orange. A/California/04/2009 HA taken from PDB structure 5GJS. A/Hong Kong/1/1968 HA taken from PDB structure 4FQY. Epitopes were labeled based on the interacting residues of monoclonal antibodies (mAbs) CR6261, CR9114, and FluA-20 with HA.

The attachment and fusion functions of the HA glycoprotein are mediated by two domains: the globular head domain and the stem/stalk domain, respectively [7]. HA is cleaved from a HA0 precursor into the HA1 and HA2 subunits, which is required for membrane fusion activity [10]. During influenza attachment, the RBS of the head domain attaches to sialic acid receptors, followed by endosomal uptake of the virus particle [10]. Low pH within the endosome triggers membrane fusion, involving insertion of the fusion peptide into the endosomal membrane and uncoating of viral genome segments [10,11]. As the predominant surface glycoprotein, HA is targeted extensively by B cells; likewise, HA-specific antibodies predominate the humoral immune response. Whereas antibodies binding the RBS or other antigenic sites/epitopes in the immunodominant, variable head domain comprise most of the response, a subset of antibodies bind to the more conserved, albeit immunosubdominant, stalk domain [12,13]. The major epitopes on the HA head for the H1 subtype are the Sa, Sb, Ca1, Ca2, and Cb antigenic sites, which were determined through viral mutagenesis studies in the presence of anti-HA antibodies [14]. Similar work using competition assays was also performed for the H3 subtype to characterize antigenic sites A, B, C, D, and E [15,16,17]. These antigenic sites comprise the apical, membrane-distal region of the HA1 head domain, including the RBS, as well as the region near the head-stem interface [14,15,16,17] (Figure 1).

## 3. Challenges with Current Seasonal Vaccines

An individual’s history of influenza exposure plays an essential role in the extent of protection to currently circulating viruses. The idea of original antigenic sin (OAS) posits that the initial exposure to a given strain imprints antibodies against certain epitopes that then dominate subsequent exposures to secondary strains [18]. Since the doctrine of OAS, related ideas of antigenic seniority and serological imprinting, where secondary exposures to antigenically drifted strains generate novel antibodies, have been proposed [19,20]. Annual vaccination is required due to variation in circulating strains and the relatively short protective effects of the antibody response post-vaccination, particularly among older individuals and those with underlying medical conditions [21,22]. Trivalent or quadrivalent vaccines, which contain two IAVs, one H1N1, one H3N2, and one or two influenza B viruses, exhibit roughly 50% to 60% vaccine coverage globally [22,23]. At present, seasonal vaccines are comprised of a combination of influenza virus strains that are predicted to be antigenically similar to those circulating at the time of vaccination [24]. As six months are typically needed from the selection of vaccine strains through vaccine production, release, and distribution, vaccine mismatch may occur due to the emergence of novel strains [23]. Moreover, differences in glycosylation between HA proteins in vaccines and those in circulating viruses can exacerbate vaccine mismatch [25,26]. Variation in the effectiveness of the current influenza vaccine can also be attributed to the rate at which influenza viruses undergo antigenic drift [27]. Antigenic drift results from the error-prone nature of the viral RNA-dependent RNA polymerase, resulting in the accumulation of mutations within the viral genome with successive rounds of replication [27].

Avian and swine transmission events also contribute to the emergence of novel influenza virus strains within the human population [27], and both natural and immune pressures on the HA select for drift variants [28]. In general, H1N1 viruses undergo antigenic drift to a lesser extent than H3N2 viruses, with approximately 2.45 amino acids substituted in the HA per year for H1N1 viruses, compared to 3.6 amino acid substitutions per year for H3N2 viruses, possibly due to the antigenically novel nature of the pandemic-like H1N1 virus [29,30]. Antigenic shift can result from reassortment events of HA genes between genome segments, such as between human and avian viruses [31]. Alternatively, zoonotic spillover events from antigenically exotic viruses can also lead to antigenic shift [31].

**Figure 2:**
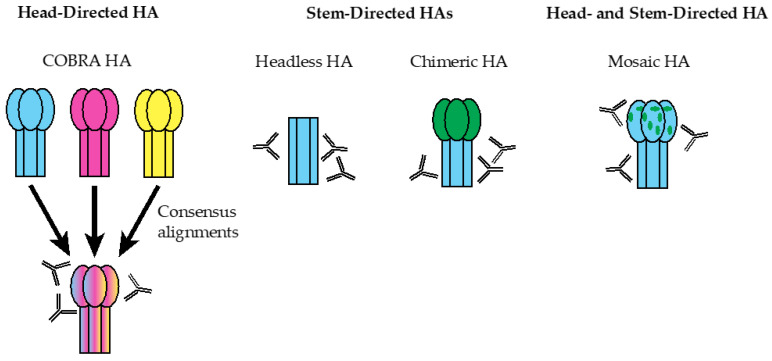
HA-based universal influenza vaccine designs. COBRA (computationally optimized broadly reactive antigen)-based HAs are head-focused HA immunogens incorporating multiple HA sequences of distinct virus strains (shown as HAs of different colors) of a particular subtype into a consensus COBRA HA (shown as a merged HA of multiple colors) that elicit mainly head-targeting antibodies. Headless HAs lack the globular head domain and are comprised solely of the stem domain to focus antibody responses to the otherwise immunosubdominant, but conserved, stem. Chimeric HAs (cHAs) consist of the globular head domain of one subtype (shown in green) and the stem domain of another subtype (shown in cyan) to be targeted, more closely mimicking a native HA molecule while focusing antibody responses to the conserved stem through exposure to multiple cHA immunogens. Mosaic HAs (mHAs), which can be seen as a refinement of cHAs, consist of the majority of the head domain and the entire stem domain of one subtype (shown as a blue HA trimer), but the head antigenic sites of another subtype (shown as green regions in the HA head), eliciting both head- and stem-directed antibodies.

The population to be immunized with the seasonal vaccine also plays a role in vaccine effectiveness and efficacy. For instance, vaccine uptake in immunocompromised and pediatric populations varies widely. For this population, adjuvanted vaccines containing MF59 and high-dose inactivated virus vaccines are available but nonetheless present an obstacle to vaccine-based protection [21,32]. To circumvent these issues, several HA-based vaccine designs have been developed and studied in preclinical studies and clinical trials (Figure 2).

## 4. Alteration of Glycosylation Sites in HA Immunogens

It has been well-documented that changes in the glycosylation patterns in the HA immunogen can greatly alter antibody reactivity. One study showed that the addition of glycosylation sites to the H5N1 virus A/duck/Niger/2090/2006 altered viral growth properties, enhancing viral diffusion due to reduced HA activity and enhanced NA activity, and also decreased the neutralizing activity of sera from vaccinated mice, thereby contributing to immune escape [33]. Similarly, H1N1 subtype influenza virus containing a glycosylation site at position 144, corresponding to antigenic site Sa, effectively masked a highly targeted site by HAI antibodies in mice [34].

It could be inferred that such glycan masking of HA epitopes, as well as the presence of non-native glycosylation sites from egg-based vaccine production, could restrict the breadth of antibody protection. Likewise, removal of these glycans, either through enzymatic means or through the use of alternative cell culture systems, to generate less-glycosylated HAs could afford enhanced breadth of protection. Studies investigating this hypothesis have demonstrated success in achieving wider protection and cross-reactivity by utilizing monoglycosylated and alternatively deglycosylated HA immunogens [34,35,36,37]. Specifically, treatment of embryonated chicken eggs with kifunensine, an inhibitor of α-mannosidase I-mediated glycosylation, in embryonated egg-based vaccine production, and virions with endoglycosidase H to trim their glycans to a monoglycosylated form, produced vaccines with superior HAI and neutralizing titers in mice [37]. Moreover, the same treatment afforded improved stem-specific antibody titers as well as antibody-dependent cellular cytotoxicity (ADCC) [37].

More subtle alterations to the glycosylation landscape of HA can alter the inter-group specificity and breadth of antibodies resulting from vaccination. Utilizing a H1 stem nanoparticle vaccine, it was shown that the introduction of a glycosylation site at position 38 in the HA1 subunit to mimic a group 2-specific stem glycosylation site changed its antigenicity, preventing the binding of group 1-specific antibodies [38]. This alteration also mediated cross-group reactivity, producing appreciable heterosubtypic neutralization against group 2 viruses and passive protection from a group 2 virus, A/Anhui/1/13 [38].

## 5. HA Head-Targeting Vaccine Designs

During the course of infection or vaccination, antibodies against the immunodominant globular head domain are predominantly elicited. Although the head is antigenically variable, there are nonetheless regions that remain fairly conserved; notably, cross-strain- as well as cross-group-reactive antibodies targeting the RBS have been well-characterized [39]. Other conserved head epitopes have also been discovered, including the ‘lateral patch’ on the lower side of the head domain [40], as well as within the HA trimer interface [41,42,43,44] (Figure 1). Antibodies binding at the intratrimeric epitope are protective but non-neutralizing. It is thought that they elicit protection through Fc receptor- and complement-dependent mechanisms [41,44], as well as by dissociating the HA trimer [41,42]. Notably, an intratrimeric epitope has recently been discovered for another type I fusion protein, the human metapneumovirus fusion protein, and an antibody targeting this epitope was neutralizing [45]. Recent strategies to target these head epitopes have gained traction and have primarily involved immunization with a ‘consensus’ HA immunogen representing multiple viruses from distinct antigenic spaces.

Computationally optimized broadly reactive antigens (COBRAs), which utilize merged sequences from divergent virus strains, represent promising vaccine candidates that are now in the late preclinical stage of development [46]. The COBRA platform is also highly amenable to several formulations, and protective effects have been noted for nanoparticle, live-attenuated, virus-like particle (VLP), and split-inactivated vaccines [47,48,49]. Each COBRA antigen represents a single viral subtype encompassing several time periods in multiple antigenic spaces using a layered consensus-building approach. Consequently, the resulting COBRA immunogens represent both the sequences and structural conformations of its constituent HAs [50]. For instance, H3N2-based COBRAs have been developed that retain the structural characteristics of its constituent sequences, including antigenic sites and glycosylation sequences [48]. Moreover, the period of time in which a set of HA sequences is selected for a given COBRA design alters the breadth of the resulting antibody response. For example, COBRAs representing a particular subset of H3N2 sequences between 1968 and 2013 were shown to elicit significantly broader HAI responses than for those where all H3N2 HAs from this period were represented [48].

A primary correlate of protection that COBRA vaccines aim to elicit is HAI activity from antibodies that bind the head domain. In this respect, COBRA vaccines have shown notable success, where leading COBRA antigens elicit significant HAI antibody titers. This has been shown for several subtypes, including H5N1 [51], H3N2 [48,49], and H1N1 [49] viruses, where such HAI antibodies likely provide protection. It is possible that COBRA vaccines target conserved regions within the head domain, and COBRA-reactive antibodies block HA attachment to sialic acid receptors through binding the RBS and nearby epitopes. Alternatively, COBRA HAs may also elicit a polyclonal antibody response targeting diverse, variable epitopes, that synergistically confer protection. In a preimmune model where ferrets were pre-infected with historical H1N1 viruses, then immunized with a H1 COBRA VLP, stem antibodies did not consistently ameliorate viral replication following challenge with A/California/07/2009 [52]. This finding suggests that stem-based antibodies are not a major mode of the protective efficacy of this vaccine design. Similar findings were shown in H3N2-based COBRAs on the basis that neutralization and HAI titers correlated with one another well following vaccination [48].

## 6. HA Stem-Targeting Vaccine Designs

The stem domain of HA is highly conserved and has shown promise as an effective immunogen. Broadly reactive stem-binding antibodies are prevalent within human sera for group 1 viruses, and stem-targeting B cells can be expanded upon exposure to the antigen [53]. Furthermore, because of its relatively conserved nature, the stem epitope elicits broad, heterosubtypic antibody protection, even across diverse phylogenetic groups of influenza virus [54,55,56]. Since the discovery of the stem epitope (Figure 1), many groups have engineered a number of HA stem immunogens to redirect the antibody response away from the more variable head domain to this conserved region of HA. These include headless HAs that completely remove the head domain, and chimeric HAs (cHAs), which replace the native head domain with an antigenically distant head from another influenza subtype.

### 6.1. Headless HA Vaccines

Headless HA vaccines are comprised solely of the HA stem domain while lacking the globular head domain, thereby overcoming head domain immunodominance. Such vaccines were initially shown to be protective in mice, where a stem-truncated HA construct protected 70% of vaccinated mice from lethal challenge of H2N2 subtype virus A/Okuda/57 [57]. Further modifications to optimize and stabilize the immunogen through the inclusion of a linker between the N- and C-termini of the HA1 subunit and incorporation into virus-like particles were successful in reducing morbidity in challenged mice and conferring cross-reactivity to viral subtypes H1, H2, and H5 [58]. However, in the same study, mouse antibody responses were limited in their cross-reactivity to intra-group subtypes [50]. In another study, Tni insect cells were used to generate VLPs co-expressing headless HA and the influenza M1 protein from H1N1 PR8 [59]. Vaccination of mice in a prime-boost-boost regimen led to increased ADCC activity, lung and nasal IgG and IgA endpoint titers, and IgG-producing antibody-secreting cells, suggesting that stem-binding antibodies were indeed being produced; however, the breadth of the immune response was not tested in this study [59].

### 6.2. Chimeric HA Vaccines

cHAs utilize a similar approach in obscuring antibody responses to the HA head domain; however, in this strategy, an antigenically novel globular head domain from one IAV is grafted onto the stem domain of interest from another IAV subtype. Multiple immunizations with cHA constructs containing similar stems but distinct head domains restrict the elicitation of novel head-directed antibodies, focusing the response to conserved stem epitopes. The rationale behind cHAs is based on observations involving primary infection with one subtype of a group 1 or 2 virus, followed by a secondary infection with a different strain of the same group with a substantially distinct head domain [60]. Sera from patients that were seropositive against a H3N2 virus showed significant increases in neutralizing activity against a cH7/3N3 influenza virus (with the H7 head and H3 stem) following infection, illustrating functional, neutralizing stem-directed antibodies against the conserved group 2 stem [60].

To further study the efficacy of the cHA approach, mice were primed with a cHA construct containing a H4 head and a H3 stalk (cH4/3 cHA), followed by boosts with a cH5/3 HA and a cH7/3 HA, and then challenged with a heterologous H3N2 virus [61]. These animals were protected from mortality following challenge [61]. Similar experiments with initially sublethal infections of virus, followed by priming with similar chimeric constructs to simulate subclinical infections in humans, enhanced levels of broadly protective antibodies against heterosubtypic viruses [61]. Protection has been established using these cHAs in ferrets with group 1 viruses, as shown by stem-specific antibodies that confer heterologous and heterosubtypic protection [62]. Most recently, cHAs were approved for a phase I clinical trial, where participants were primed with live-attenuated or inactivated cH8/1N1 virus, then boosted with cH5/1N1 inactivated vaccine [63]. Encouragingly, some potentially protective HAI and neutralizing antibodies appeared to be elicited, alongside broadly reactive, stem-directed antibodies [63]. Interestingly, structural analysis of a cH5/1 cHA (containing a H5 head and a H1 stem) showed that, compared to the native HAs of the constituent head and stem subtypes, the head of the cHA is misplaced on the stem by 60 degrees, while still retaining functionality in viral entry and antigenicity in the stem and head epitopes [64]. The fact that these properties are retained despite structural differences in the cHA conformation suggests that HA is relatively plastic and can accommodate such differences while retaining robust immunogenicity.

cHAs immunogens have also been shown to be protective in mice when combined with stem-only immunogens. In an immunization regimen involving priming with a cH9/1 cHA (containing a H9 subtype head and a H1 subtype stem) DNA vaccine, followed by two boosts with a PR8-based H1 headless HA, complete protection from homologous H1N1 virus and moderate protection from heterosubtypic H5N1 and H6N1 viruses were achieved [65]. Notably, headless stem immunogens from this study did not appear to induce traditional neutralizing activity as the conformations of neutralizing epitopes differed from those of full-length HA [65]. To overcome this issue, ‘mini-HAs’ were engineered in a study based on the HA of the H1N1 A/Brisbane/59/2007 virus, now aiming to maintain its native trimeric conformation [66]. These constructs were quite immunogenic in mice, and the resulting antibodies bound the full-length HA of the homologous virus [58]. Moreover, these antibodies also showed neutralizing and ADCC activity [66]. Non-human primate models also showed neutralizing stem-directed antibodies following immunization with these constructs [58]. These antibodies also demonstrated broad cross-group binding and heterosubtypic neutralization [66]. Similar heterosubtypic protection has also been found in a nanoparticle-based platform, in a mechanism that may rely on Fc effector functions rather than neutralization [67].

## 7. HA Head- and Stem-Targeting Vaccine Designs

Both neutralizing head-targeting antibodies and broadly reactive stem antibodies are likely necessary for an optimal immune response to influenza. Likewise, approaches that elicit both types of antibodies would be ideal in conferring robust protection.

### Mosaic HA Vaccines

Mosaic HAs (mHAs) utilize a HA immunogen that is a composite of several HA sequences. In one approach, the whole stem domain and head domain of one subtype is merged with the major antigenic sites of another subtype or genus to overcome strain-specific responses while retaining conserved and neutralizing epitopes [68,69]. In one study, mice were primed with the H4 subtype HA in a DNA vaccine, followed by two boosts with inactivated viruses expressing cHA constructs, one with a mH10/3 HA, then a mH14/3 HA, comprising antigenic sites A through E of either the H10 or the H14 subtype, respectively, and the remaining head and stem residues from the H3 subtype [68]. This approach elicited antibodies with Fc-mediated effector functions targeting the stem domain, in addition to neutralizing, head-directed antibodies [68]. These results suggest that the mHA approach can elicit both effective anti-head antibodies like those produced by the current vaccine, as well as broader anti-stalk antibodies similar to those produced from chimeric and headless HAs.

Another mHA approach, utilizing an immunogen representing the H1 subtype from 1918 to 2018 viruses, employs combined sections of full-length HA sequences to generate a novel HA while retaining conformationally important features predicted to be necessary for antibody binding [70]. Originally derived from HIV vaccinology approaches to broaden the immune response against mismatched strains, this mHA was effective in eliciting antibodies against divergent H1 strains [70,71]. One mHA immunogen showed close sequence similarity with pre-pandemic strains, including A/Brisbane/59/2007 [70]. In vivo studies of vaccination using an Ad5-vectored antigen confirmed the presence of antibodies with broader HAI activity against pre-pandemic H1 viruses [70]. High antibody titers were detected by ELISA, but only a fraction had HAI activity, suggesting the presence of stem antibodies [70]. Studies on the extent to which such stem antibodies are produced from mHA immunization are warranted. Other groups utilized the same approach, showing its efficacy in eliciting neutralizing, homosubtypic protection through DNA and recombinant protein formulations, as well as heterologous protection in modified vaccinia Ankara formulations, illustrating versatility in the formulation method while retaining protectiveness [72,73].

## 8. Adjuvant Effects on Vaccine Responses

Adjuvants are commonly used in inactivated and recombinant vaccines to stimulate a more robust immune response akin to that of live-attenuated vaccines. Adjuvants also provide other beneficial effects, including antigen dose-sparing [74], the induction of a preferentially biased immune response, and enhancing antigen immunogenicity [75]. Some currently licensed influenza vaccines already include adjuvants, such as MF59, a squalene-based oil-in-water emulsion, which is present in the Fluad vaccine aimed at individuals aged 65 and older; AS03, another oil-in-water adjuvant, is also used in licensed influenza vaccines in Europe, such as Pandemrix [75,76,77]. The ongoing discovery and design of several adjuvants, along with the elucidation of their mechanisms of action, are particularly relevant for the influenza vaccine where a robust humoral response is now known to be essential for protection. Adjuvants are especially useful for inducing robust antibody responses in high-risk populations, such as the elderly and those with pre-existing conditions such as HIV and obesity. Below we summarize the properties of common and novel adjuvants and their impacts on the influenza vaccine response.

### 8.1. Aluminum Salts and Alum

Alum, the oldest adjuvant in use, includes a range of aluminum salts such as aluminum hydroxide and is also the most widely used adjuvant in humans. It is currently included in several vaccines, such as the DTaP and hepatitis A and B vaccines [77], and is known to provide a strong, Th2-skewed response characterized by IgG1 antibodies [78,79]. Moreover, HAI titers were significantly increased in its presence compared to no adjuvant during subcutaneous influenza immunization with subunit HA antigen [78]. Although alum induces a strong Th2-biased immune response and HAI titers, this may not necessarily correlate with virus clearance. In mice vaccinated with PR8 H1N1 whole inactivated virus (WIV), IgG1 antibodies increased while IgG2a antibodies decreased, typical of a Th2 response [79]. In addition, the lung viral titers in mice receiving alum-adjuvanted WIV were nearly two logs higher than in mice receiving WIV only [79]. This may illustrate the necessity of stimulating a less Th2-polarized, more mixed Th1/Th2, or Th1-polarized response to gain a more protective IgG1/IgG2a ratio that alum alone cannot provide, at least with a WIV subunit vaccine.

### 8.2. Oil-in-Water Emulsions

MF59 is used in current influenza vaccines and elicits a broadly reactive B cell response, dependent in part on the induction of strong immune memory [80,81]. This adjuvant can stimulate both cellular and humoral responses, and when compared to alum, can induce similar, if not higher, HAI and antibody titers [78,82]. Furthermore, a major advantage of MF59 is in inducing a protective immune response from vaccination for at-risk populations, including young children and in the elderly, eliciting higher HAI titers when adjuvanted [83,84,85]. The mechanism by which MF59 provides superior antibody protection has yet to be fully characterized, although one study suggested the elicitation of cross-reactive antibodies in a prime-boost regimen for H5N3 viruses [80]. In another study, the antibody epitope repertoire in both adults and children was found to be diversified for those receiving a MF59-adjuvanted, inactivated 2009 pandemic vaccine compared to a non-adjuvanted control [86]. Serum antibodies binding the H1 HA1 subunit were significantly increased, had higher affinity, and correlated with higher virus neutralization in individuals receiving the MF59-adjuvanted vaccine [86]. Interestingly, MF59 appeared to shift the pool of antibody epitopes towards the head domain, away from the HA2 stem domain, and also increased affinity maturation against a novel H5N1 strain following initial H1N1 exposure [86]. More recently, the role of antibody effector functions has been implicated in the mechanism of MF59. In one study, it was found to enhance complement deposition and neutrophil phagocytosis, but not antibody-dependent monocyte or NK cell effector functions, suggesting a more complex role of Fc receptors and complement beyond the traditionally accepted roles of Fcγ receptors [87].

AS03, a similar squalene-based adjuvant, has been used in current influenza vaccines for its capacity to produce high antibody titers and increase breadth of protection [88]. Similar to MF59, AS03-adjuvanted animals receiving a split-inactivated H5N1 vaccine produced high levels of neutralizing antibodies against homologous and heterologous H5N1 viruses in ferrets [89]. Individuals receiving TIV followed by AS03-adjuvanted pandemic H1N1 (pH1N1) HA showed enrichment for plasma cells with mutated BCRs that cross-react with pH1N1 HA; furthermore, naïve B cells were also more strongly activated when adjuvanted, and had increased isotype switching to IgG1 and IgG3 [90]. Adjuvanted vaccines also altered the proportion of V gene alleles that were utilized and mutated in BCRs; notably, mutations in the *V_H_1-69* allele, associated with stem-binding antibodies, comprised a higher part of the total repertoire when the pandemic vaccine was adjuvanted with AS03 [90]. Although these results suggest a similar mechanism of AS03 to that of MF59, further studies to confirm correlations between BCR sequences and antibody epitopes are warranted. Also similar to MF59, AS03 appeared to play some role in stimulating complement-dependent lysis (CDL) for individuals receiving the 2009 pH1N1 vaccine [91]. Furthermore, this CDL activity extended to a small extent to a heterologous, pre-pandemic influenza virus strain [91].

### 8.3. TLR Agonists

Toll-like receptors (TLRs) are proteins present ubiquitously on external and internal membranes of certain immune cells, and recognize pathogen-associated molecular patterns (PAMPs). Downstream signaling pathways transduce ligand binding, producing immune activation phenotypes that can be modulated based on the ligand (and thereby the TLR) [92]. A number of TLR agonists have been engineered for potential use in influenza vaccines. One such TLR agonist, 3M-052, an imidazoquinoline that binds TLR7/8, has been shown to broaden the antibody response in pandemic H5N1 HA antigen vaccination in ferret and mouse models [93]. Immunization of ferrets with a split vaccine paired with this adjuvant provided protection from homologous and heterologous drifted strains, which may be due to an increase in V gene diversity, a result that was previously observed for co-administration of the adjuvant with malarial antigen [93]. Further studies are necessary to determine whether heterosubtypic neutralization is observed. In another study, the A/California/7/2009 HA globular head domain was fused to bacterial flagellin, a TLR5 agonist [94]. Increases in HAI titer and seroconversion were seen for both young (18–49 years old) and old (65 years or older) populations, showing appreciable seroprotection and seroconversion in the older population [94]. The fact that a robust immune response was elicited in older individuals may be attributed to the innate stimulation of TLR5 by the flagellin component/adjuvant of the vaccine antigen, showing a proof-of-principle where antigen and adjuvant are covalently linked. CpG, a TLR9 agonist, has also shown evidence of enhancing the antibody response to influenza. When CpG was conjugated to nanoparticles comprising the A/New Caledonia/20/1999 HA, ELISA binding and HAI titers were significantly increased to an extent higher than when CpG was only mixed with HA nanoparticles [95]. Another notable TLR9 agonist, CpG 1018, formulated by Dynavax Technologies, was recently shown to elicit neutralizing antibodies against SARS-CoV-2 when paired with the pre-fusion spike protein and alum, eliciting a Th1-type response [96], and has also been approved for use in a hepatitis B virus VLP vaccine (HEPLISAV-B) [96]. This CpG agonist in the influenza vaccine may be useful in amplifying similar neutralization-based protection.

### 8.4. Advax

Advax has shown a promising safety profile when used in split-virion and recombinant influenza vaccines [97,98]. As a delta inulin microparticle-based polysaccharide adjuvant, Advax is comprised of 1–2 µm particles, and has also been investigated for intranasal vaccinations and as a mucosal adjuvant [99]. Immunization of mice with WIV and Advax adjuvant increased lung IgG antibody titers, as well as IgG and IgA antibody-secreting cell populations compared to the non-adjuvanted group; Advax also increased the memory B cell response [99]. A single high dose of Advax-adjuvanted, inactivated vaccine has been shown to improve B cell responses in neonatal mice, leading to increased class-switching from the IgM to the IgG1 isotype following vaccination with inactivated H1N1 and Advax [100]. Therefore, Advax may be an effective adjuvant to include in pediatric populations that receive the influenza vaccine to improve memory and class-switching responses for inactivated formulations. Whether the adaptive response to an Advax-adjuvanted vaccine is Th2- or Th1-biased appears to depend on the antigen used, where the split-inactivated vaccine adjuvanted with Advax induces a Th2-type response, whereas WIV adjuvanted with Advax induces a Th1-type response [101].

### 8.5. Iscomatrix

Iscomatrix adjuvant consists of cage-like structures comprised of phospholipid, saponin, and cholesterol; these structures promote a balanced Th1/Th2 response and antigen trafficking into the lymph nodes, as well as the production of intracellular antigen depots within dendritic cells for sustained presentation [102]. When used with H7N9 VLPs to immunize mice, homologous protection from a lethal challenge was achieved, in addition to protective HAI titers against both homologous H7N9 and heterologous H7N3 [103]. Similar to MF59 adjuvant, epitope spreading of higher-affinity antibodies against the HA1 subunit was also observed for individuals receiving a H7N9 VLP vaccine with Iscomatrix adjuvant, possibly resulting from increased germinal center reactions of HA-specific T cells with B cells [104]. The interaction of T and B cells may drive increased receptor affinity as well as novel stimulation of clones reactive against HA1 [104]. Furthermore, the off-rates of antibodies binding HA1 were significantly lower when the VLP vaccine was adjuvanted, and a negative correlation was seen between the heterologous dissociation rates of serum antibodies to vaccine strain H7 HA1 and the neutralizing titers to a heterologous H7 virus [104].

## 9. Conclusions

HA head- and stem-directed vaccine designs are currently under investigation to improve upon current influenza vaccines. Despite the variability of influenza virus, conserved epitopes have nonetheless been identified through antibody epitope analysis. The COBRA approach has proven the ability to elicit potent antibodies with HAI and neutralizing activities that likely target a diverse number of epitopes on the globular head domain. This consensus layering approach aims to target sequences that will be present in future pandemic and seasonal HA sequences. In contrast, stem-directed designs have shown success in narrowing the antibody response to the relatively conserved stem domain, now having elicited stem antibodies in a phase I trial utilizing the cHAapproach. Headless HA designs have also been refined greatly, preserving the natural conformation of native HAs and its associated epitopes. mHA vaccines appear to elicit antibodies against both the HA head and stem domains, potentially optimizing the antibody response to maintain a neutralizing and broadened epitope pool. Further studies into the impact of pre-existing immunity in these vaccine approaches may inform the role of original antigenic sin in adopting a universal influenza vaccine. Considering that vaccine immunogen design efforts have been historically biased towards HA, recent studies into next-generation NA immunogens, the more conserved surface glycoprotein, have also shown promise, and optimal breadth of protection may only be achieved through the combination of next-generation HA and NA immunogens [105,106,107]. Adjuvants that have also been employed for use in current and next-generation influenza vaccines, such as alum, AS03, and MF59, and more novel systems like TLR agonists, Advax, and Iscomatrix, are only now beginning to be understood for how they might alter the antibody response. The discovery that they broaden the antibody repertoire may be key to optimizing the elicitation of broadly neutralizing antibodies during vaccination, and further studies to illuminate this aspect are certainly needed. Overall, continued studies into these two components will be essential to develop a universal influenza vaccine.

## Data Availability

No new data were created or analyzed.
